# Interrepetition Rest Set Lacks the V-Shape Systolic Pressure Response Advantage during Resistance Exercise

**DOI:** 10.3390/sports5040090

**Published:** 2017-12-01

**Authors:** Xian Mayo, Eliseo Iglesias-Soler, J. Derek Kingsley, Xurxo Dopico

**Affiliations:** 1 Active and Healthy Lifestyle Observatory, Centre for Sport Studies, King Juan Carlos University, Madrid 28942, Spain; 2 Performance and Health Group, Department of Physical Education and Sport, Faculty of Sports Sciences and Physical Education, University of A Coruna, A Coruña 15179, Spain; eliseo@udc.es (E.I.-S.); xurxo.dopico@udc.es (X.D.); 3 Cardiovascular Dynamics Laboratory, Exercise Physiology, Kent State University, Kent, OH 44242, USA; jkingsle@kent.edu

**Keywords:** pressure response, aneurysm, strength exercise, set configuration

## Abstract

Resistance exercise may lead to an aneurysm due to dangerous levels of systemic hypertension. Thus, a minimized pressure response during exercise may guarantee safer training. For that, we analyzed an interrepetition rest design (IRD) hypothesizing that it would produce a lower systolic blood pressure (SBP) response in comparison with a continuous design (CD). Additionally, we studied the effect of accumulated repetitions on the increasing SBP rate during the first continuous set. Fifteen healthy participants (age: 24 ± 2 years; SBP: 113 ± 8 mmHg) performed leg presses, with 40 repetitions and 720 s of total rest, structured in an IRD of individual repetitions (resting time: 18.5 s), and in a CD of five sets of eight repetitions (resting time: 180 s). Analyses reported an increase (*p* = 0.013) in the mean peaks of SBP in the IRD (162 ± 21 mmHg), versus the CD (148 ± 19 mmHg), while both augmented versus baselines (*p* < 0.001). Additionally, the linear model estimated a progressive increase of SBP of around 7 mmHg per repetition. Summarily, the IRD produced a higher mean of the SBP peaks during the 40 repetitions due to lacking the v-shape advantage in comparison with the CD.

## 1. Introduction

Strength training is a promising intervention for the prevention and amelioration of several pathologies, and leads to a reduced all-cause mortality risk [1]. Nevertheless, an acute bout of resistance exercise may lead to a catastrophic occurrence due to artery dissection [2]. This is a consequence of the vascular stress provoked by the extreme pressure response that occurs during strength training [2]. Despite the fact that artery dissection is a rare condition, systemic hypertension may yield extremely dangerous levels of wall stress, in some cases reaching the breaking point of the wall strength. This might occur particularly in individuals with a pre-existing enlargement of an artery [3]. However, strength training provides unique health benefits [4], which outweigh its possible risks, and is usually safe for the general population [5].

The pressure response in dynamic resistance exercise has been studied traditionally using the leg press exercise as a model [6]. During a leg press set, there is an excessive pressure response during the first repetition [6,7,8]. This is the consequence of mainly four factors. Firstly, the feet elevation from the resting to the exercise position [7]. Secondly, the isometric contraction during the position adjustment before the beginning of the exercise [8]. Thirdly, the effort performed to overcome the absence of elastic energy stored in the muscles [8]. Fourthly, an excessive intrathoracic pressure that is the consequence of all the previous issues [6]. During the next few repetitions, there is a v-shape pressure response. This occurs simultaneously with an enhanced use of the elastic energy, which is stored in the muscles in an absence of fatigue [8,9]. The v-shape pressure response is a hemodynamic advantage, since some repetitions can be performed without an elevation of the blood pressure [8,9]. Once the fatigue begins and increases, there is a progressive increment in the systemic pressure response. This originates reflexively in the active muscles due to the mismatch between blood flow and metabolic demands [10]. There is also an augmenting intrathoracic pressure due to the Valsalva maneuver [6]. The junction between both causes leads to a peak pressure response, which occurs at the last repetition, before the occurrence of muscular failure [6].

The Valsalva maneuver is particularly performed when lifting heavy loads, or when light loads are lifted to failure. In a situation of muscular disadvantage [6], the maneuver pressurizes the abdominal cavity, which aligns the trunk better, and creates a rigid compartment, which facilitates the application of force to the reel of the leg press machine. Because of the exaggerated pressure response that occurs, the maneuver is commonly discouraged by coaches [11]. Nevertheless, when the Valsalva maneuver is attempted, the pressure response is somewhat attenuated, but never truly dampened [12]. In fact, the rigid compartment that the Valsalva maneuver creates is needed to reduce the spinal disc compressive forces that increase. Therefore, by lifting safely, the maneuver should not be discouraged [12].

Despite the need for a Valsalva maneuver for a safer lift, an exaggerated pressure response indeed increases the transient risk of artery dissection, even in young healthy individuals [3]. Therefore, the loading parameters used in the session should be carefully selected, to guarantee the vascular integrity of the trainee, while maximizing the benefits of strength training. In this sense, convenient strategies to reduce the pressure response include selecting a rational number of sets and a sufficient break between sets [13], and choosing a reasonable compromise between intensity of load and the length of the set with that particular load [8].

In relation to the latter, it was previously suggested that the pressure response, occurring with a particular intensity of load, is more affected by the length of the set performed, than the intensity of the load, per se [8,14]. Thus, protocols such as the interrepetition rest design (IRD), allowing a lower glycolytic involvement, as a consequence of the reduction in muscular fatigue [15], might be an attractive option, compared to continuous designs (CD). This is because IRD prevents the exacerbated increase in the pressure response, when one is close to muscular failure. Nevertheless, muscular failure would occur with a preeminent intrathoracic pressure response due to the start of a new set in every repetition [6]. On the other hand, CD has a higher glycolytic involvement due to muscular fatigue, which may considerably increase the reflex pressure response [10]. However, at the same time, CD might benefit, in part, from the v-shape pressure response advantage. One can perform the repetitions, which occur in the set, without muscular fatigue [8].

Three reports previously studied this particular topic [7,16,17], but probably the resting time (2–10 s) between repetitions, or groups of repetitions in the short sets, was not enough to maintain the phosphocreatine muscle content [9], thus failing to avoid the involvement of the glycolytic metabolism when comparing it with continuous sets. In our study, we aimed to compare the pressure response of an IRD, with enough resting time of a CD. Additionally, we aimed to understand the progression of the systolic blood pressure (SBP) in a CD to estimate the effect of accumulated repetitions on the increasing rate of SBP throughout the set. Our inference is that the IRD would have a lower pressure response in comparison with the CD, as a consequence of a lower glycolytic involvement, despite the lack of the v-shape pressure response advantage. Additionally, we also hypothesized that the increase of SBP throughout a set can be estimated based on the accumulated number of repetitions.

## 2. Materials and Methods

### 2.1. Participants

Fifteen healthy participants (10 men and 5 women) with at least six months of weight-lifting experience volunteered (age: 24 ± 2 years; height: 1.74 ± 0.08 m; body mass: 67.7 ± 9.0 kg; resting heart rate (HR): 58 ± 13 bpm; SBP: 113 ± 8 mmHg; diastolic blood pressure (DBP): 66 ± 7; 10-repetition maximum on leg press (10RM): 211 ± 37 kg). All participants were screened and excluded if they had a previous history of cardiovascular disease, or were taking any medication or substance that could potentially affect the results. Participants signed an informed consent and were suitably informed about their rights. The research project obtained the formal ethical approval by the University of A Coruña and was in full compliance with the Declaration of Helsinki.

### 2.2. Procedures

Participants attended seven different days in total at the same time (±1 h), separated by a 72 h lapse. Before testing, they were asked to refrain from alcohol, caffeine, nicotine, and exercise for 24 h, and to fast for three hours. Participants underwent three orientations, two testing and two experimental sessions. In all sessions, warm-ups consisted of five minutes of submaximal treadmill exercises at 70–90% of the estimated maximum HR, five minutes of joint mobilization, and 2 sets of 10 repetitions with the 50% of the 10RM load.

#### 2.2.1. Orientation and Testing Sessions

In the orientation sessions, participants were instructed on how to perform the leg press exercise, and to learn testing procedures to obtain appropriate, quality hemodynamic values throughout the experimental sessions. The leg press was performed in an eccentric–concentric fashion using a diagonal sled-type machine (Biotech Fitness, São Paulo, Brazil). Participants were asked to push the reel from the lock to the position in which they had their knees fully extended, and then to start the movement. They were asked to lower the weight in a controlled manner, until reaching 90° of flexion in both hip joints and knees, touching the knees with a rubber band. After reaching this position, participants had to return to the position of full knee extension, performing the repetitions at the maximal intended velocity. 

In the testing sessions, 10RM testing was performed as the load that participants could lift no more than 10 times, with appropriate form and without rest between repetitions [18]. Experimental sessions were prescribed with the second testing load.

#### 2.2.2. Experimental Sessions

**Figure 1:**
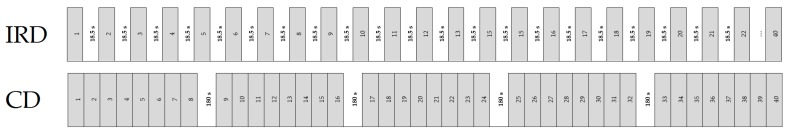
Schematic representation of the experimental sessions. At the top, the interrepetition rest design (IRD). At the bottom, the continuous design (CD).

The two experimental sessions were performed in a randomized order. Both sessions consisted of performing a total of 40 repetitions with 720 s of total rest, at the 10RM load. Therefore, the work-to-rest ratio was equated between sessions. The IRD consisted of 40 individual repetitions with 18.5 s of rest between repetitions, with the intensity of effort at 10% (i.e., 1 repetition performed out of 10 feasible repetitions). The CD consisted of 5 sets of 8 repetitions with 180 s of rest between sets, with the intensity of effort at 80% (i.e., 8 out of 10). A schematic representation of the experimental sessions is presented in Figure 1.

#### 2.2.3. Data Recording and Analysis

Continuous three-lead electrocardiogram and beat-by-beat blood pressure (BP) were obtained using a Task Force Monitor (CNSystems, Graz, Austria). The electrocardiogram was used for obtaining HR at a rate of 1000 Hz. Finger BP on the left hand was obtained with a pneumatic cuff by photoplethysmography with a sampling frequency of 100 Hz. The arm of the pneumatic cuff was held by a sling with the finger’s cuff at the fourth intercostal space level. Finger BP was automatically and continuously transformed into brachial BP by the device with an oscillometer placed on the right arm. SBP and DBP obtained the higher and lower values of the BP trace, respectively. Additionally, pulse pressure (PP) was calculated as SBP–DBP and double product (DP) was computed as HR × SBP and reported in bpm × mmHg × 10^−2^.

Data were analysed at baseline during the last 5 min of a 10-min period and during the 40 repetitions of the experimental sessions semi-recumbent on the leg press machine. The precautions to avoid data loss were (a) placing the right arm in a supinated position on a chair to prevent grasping the machine; (b) avoidance of odd flexions or contractions, neither with the arm or the finger with the plethysmography cuff; and (c) maintenance of a normal breathing pattern, since a strong Valsalva maneuver may affect the BP collection by plethysmography; however, the Valsalva maneuver was not forbidden [19]. Data were only considered suitable for analysis when at least 80% of the total BP tracing of every session was collected. The percentage of valid data in six participants was lower than 80% in the IRD, so only nine participants (7 men and 2 women) were compared between sessions.

For every repetition, the total duration of each repetition, as the sum of the concentric and eccentric parts, was collected with a dynamic measuring device (T-Force System, Ergotech, Murcia, Spain) attached to the sled of the leg press. The total duration of each repetition was used to select the higher SBP value of that particular repetition, and the HR and DBP values belonging to the same cardiac beat. For each design, the average of the total, concentric, and eccentric time of all repetitions were analyzed as indicators of total, concentric, and eccentric time under tension (TUT), respectively [16].

### 2.3. Statistical Analysis

Descriptive statistics are shown as mean ± standard deviation. Reliability of the 10RM was tested with the intra-class correlation coefficient (ICC = 0.99). Normality was tested using the Shapiro–Wilk test. A 2 × 2 repeated measures analysis of variance (ANOVA) was used to analyze the effect of the session (i.e., IRD vs. CD) across time (baseline vs. during exercise). During exercise, the mean of the SBP peaks of the 40 repetitions (and the HR, DBP, PP, and DP of the same cardiac beat) and the peak SBP of the session were analyzed. Additionally, a separated two-way ANOVA was also used to analyze the effect of the session across time for SBP during the first (i.e., 1st, 9th, 17th, 25th, 33th) or eighth (i.e., 8th, 16th, 24th, 32th, 40th) repetition of every set in the CD in comparison with the concomitant repetition in the IRD. Pairwise comparisons were performed using Bonferroni correction and *p* ≤ 0.05 was established as significance level. A paired *t*-test was used to analyze the effect of the session on the duration of the total, concentric, and eccentric TUT of the 40 repetitions.

The prediction of the progression of SBP in the CD was performed with a one-way repeated measures ANOVA of 9 levels (baseline and the eight repetitions of the first set). Individual linear regressions of SBP on repetitions that were significantly different from the baseline after the v-shape were obtained. Thereafter, only the participants with an individual correlation higher than 0.8 between repetitions and SBP (*n* = 12) were considered to perform a linear mixed model analysis to estimate the increase ratio (i.e., slope: mmHg/repetition) and the variability of this slope between individuals. Linear mixed models are suitable for regression analysis with correlated data [20]. Thereby, three random coefficient models were fitted (Model 1: intercept and the slope as both fixed and random effects. Model 2: Model 1 plus adding 10RM load as fixed effect. Model 3: Model 1 plus 10RM × repetition interaction as a fixed effect to consider the load’s effect on the slope). From the different tested models, Model 1 was used since it was the most parsimonious (6 parameters vs. 7 in the rest) and with the best global goodness of fit as assessed by −2log likelihood test. Statistical analyses were performed using IMB SPSS Statistics 19.0 (IBM, Armonk, NY, USA).

A post hoc statistical power (1-β) of 0.9 was calculated with the G*Power 3.1.7 (University of Kiel, Kiel, Germany) for a repeated measures ANOVA with an effect size of *f* = 0.35 and *n* = 9, and *r* = 0.7.

## 3. Results

**Figure 2:**
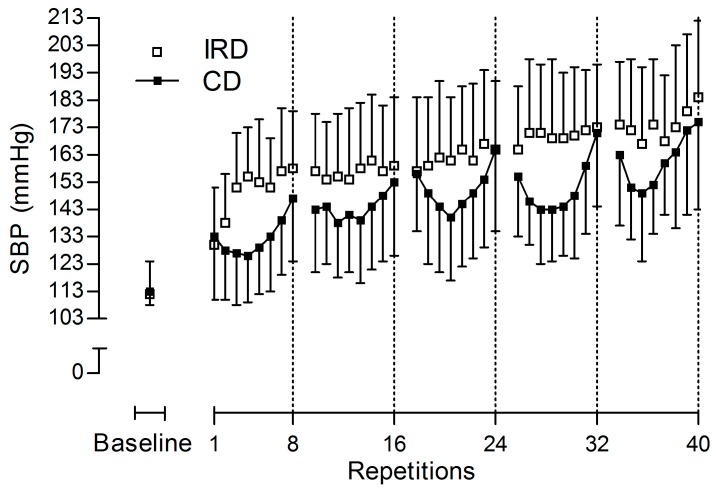
Descriptive responses of systolic blood pressure (SBP) peaks during the 40 repetitions for the interrepetition rest design (IRD, white squares) and continuous design (CD, black squares). Data are displayed as means ± standard deviation (*n* = 9).

Hemodynamic values before exercise were not significantly different between protocols for all variables (*p* > 0.05). Regarding SBP responses (Table 1), for the mean of the SBP peaks of the 40 repetitions there was a significant interaction between session and time (*p* = 0.018), such that both (*p* < 0.001) IRD and CD were higher during the sessions in comparison with the baselines, and SBP values during the IRD were higher than the values during the CD (*p* = 0.013). There was no main effect of the session (*p* = 0.052), and a main effect of time was reported (*p* < 0.001), with higher values during exercise compared with its baseline values. The progression of the SBP peaks during the 40 repetitions is descriptively reported in Figure 2.

In regard to the peak SBP of the session (Table 1), there was not a significant interaction between session and time (*p* = 0.492). Besides, there was not a main effect of session (*p* = 0.517). However, there was a main effect of time (*p* < 0.001), as peak values during the session were higher than baseline values.

For the SBP peaks of the initial and final repetitions of the five sets during the CD, in comparison with the concomitant repetitions in the IRD, there were not significant interactions between session and time either for initial (*p* = 0.194) or final (*p* = 0.564) repetitions. There was not a main effect of session either for initial (*p* = 0.274) nor for final (*p* = 0.467) repetitions. There was a main effect of time for both initial (*p* < 0.001) and final (*p* < 0.001) repetitions, with values increasing during new sets.

The rest of the hemodynamic variables are reported in Table 2. For HR, there was a significant interaction (*p* < 0.001), as both protocols increased HR during the session versus the baseline values (*p* < 0.001), but HR during the IRD was lower in comparison with the CD (*p* < 0.001). Besides, there was a main effect of session (*p* < 0.001), with lower HR values during the IRD in comparison with the CD, and a main effect of time (*p* < 0.001). DBP analysis reported no significant interaction for DBP (*p* = 0.056) and no main effect of session (*p* = 0.14). Also, a main effect of time was reported (*p* < 0.001). PP analysis indicated a significant interaction (*p* = 0.002), where PP increased during the IRD, in comparison with the CD (*p* < 0.001) and the baseline values (*p* = 0.017). A significant main effect of session was also noted (*p* < 0.001), with higher values during the IRD in comparison with the CD. Besides, a main effect of time was not reported (*p* = 0.14). Finally, for DP there was no interaction (*p* = 0.069) and no significant main effect of session (*p* = 0.097). Besides, a significant main effect of time was observed (*p* < 0.001). For HR, DBP, and DP, the main effect of time reported higher values during exercise in comparison with the baseline.

Total TUT was not significantly different (*p* > 0.05) between protocols (IRD: 1757 ± 199 ms; CD 1736 ± 242 ms). IRD was significantly shorter during concentric TUT (*p* = 0.004, 769 ± 60 ms) and significantly longer (*p* = 0.033, 988 ± 161 ms) during eccentric TUT in comparison with CD (870 ± 91 ms and 866 ± 195 ms, respectively).

**Figure 3:**
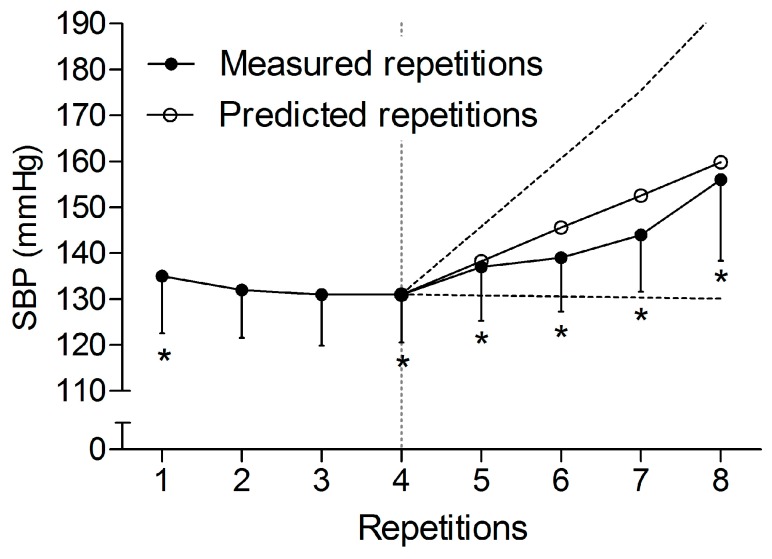
Lineal mixed model analysis of the systolic blood pressure (SBP) peaks during the fourth last repetitions of the first set of the continuous design, once the v-shape was surpassed. Black circles refer to the SBP peaks measured during the first set of the continuous design. White circles refer to the predicted SBP peaks for the fourth last repetitions. Black dashes refer to the extreme tendencies considering the estimated individual variability in the increase (slope) of SBP peaks for the fourth last repetitions. * Significantly different versus baseline values (*p* < 0.05). Data are displayed as means ± 95% confidence intervals (*n* = 12).

For the lineal mixed model analysis (Figure 3). The ANOVA test of SBP during the first set of CD revealed a significant main effect (*p* < 0.001), with pairwise comparisons showing significantly higher values in 1st and 4th–8th repetitions in comparison with the baseline (*p* < 0.001). Fitting Model 1 to these data showed a fixed effect of repetition (i.e., estimated population slope in the repetition SBP relationship) of 7.28 mmHg (standard error: 1.27; *p* < 0.001). Random effect analyses estimated a standard deviation of 3.83 mmHg, and therefore, an intersubject variance of 14.68 (mmHg/rep)^2^ for the slope of regression (7.28 ± (1.96 × 3.83)).

## 4. Discussion

There were two main findings in this study: (a) in contradiction with our hypothesis, the IRD produced an increase in the mean of the SBP peaks in comparison with the CD due to the lack of the v-shape advantage; and (b) in the first set of the CD, we observed a progressive linear increase in SBP from the fourth repetition, estimated at around 7 mmHg per repetition.

Our data showed that when the intensity of load and work-to-rest ratio are equal, set configuration defines the SBP response, as was previously suggested [8,14]. Differences between designs may be a consequence of dissimilar reflex pressor responses [10] and distinct intrathoracic pressures as a consequence of different needs on the Valsalva maneuver execution [6]. In this sense, despite the fact that we did not analyze the metabolic demands of the protocols, IRDs have a higher concentric velocity (i.e., a shorter concentric TUT), which is negatively correlated with the glycolytic involvement [21]. Since glycolytic involvement determines the reflex pressure response [10], this suggests that during the IRD, the pressor response from a peripheral origin was potentially reduced in comparison with the CD. On the other hand, an individual repetition produces an excessive intrathoracic pressure in comparison with subsequent repetitions [6], thus propagating and increasing the momentarily SBP into the next individual repetition, to a greater extent than the CD does. 

Otherwise, the CD might have a remarkable SBP response from a reflexive origin, since during the last repetitions there is an increase in the metabolic demands of active muscles [9], and an augmented intrathoracic pressure, to facilitate the application of force to the reel in a situation of muscular fatigue [6]. Nevertheless, differences between protocols only occurred during the v-shape of the CD due to an advantageous SBP response (Figure 1), which occurs simultaneously with an enhanced use of the elastic energy, stored in the muscles in an absence of fatigue [8,9]. It is important to note that differences were not observed either at the beginning or at end of every set, nor at peak SBP values of the sessions when comparing both protocols.

Our results agree with two previous studies comparing cluster set designs with continuous set designs [16,17]. These designs observed higher increases in SBP response, with two to ten seconds of resting time in the middle of the set, in comparison with the continuous ones. Oddly, our data disagree with the lower values observed in an IRD with 3 s of rest between repetitions in comparison with a CD [7]. The divergence of the results may be due to three different methodological reasons. Firstly, our leg press had an eccentric–concentric fashion in comparison with the concentric–eccentric structure of Baum et al. Secondly, in comparison with the work-to-rest ratio matched designs in our study, the protocols in the study of Baum et al. were unequal. They had the same resting time between sets but a greater total resting time in their IRD, because of the addition of the rests between repetitions, but without adjusting their CD. Additionally, while we collected the peak SBP value for every repetition, Baum et al. collected the whole SBP response (i.e., 50% resting time and 50% concentric and eccentric contractions time). These two reasons may have greatly reduced the SBP response in their study [13]. Thirdly, the times participants lifted their feet in our IRD (i.e., one for every repetition, 40 times) might have affected SBP in a greater magnitude than Baum et al.’s (i.e., three times), since after every lifting of their feet, SBP shifted upwards [7].

Peak SBP responses during our study exceeded the recommended limit of 200 mmHg to maintain aortic integrity [2]; as was previously reported, (MacDougall et al., 1992, 1985) fatal events did not occur. Regardless, it would be wise to develop protocols that minimize these peaks to achieve a safer design. Accordingly, stopping the session just after the v-shape during a theoretical CD may help to ensure the lowest values of SBP. In our study, this occurred analytically after the third repetition and descriptively after the third or fourth repetition (Figure 1). Thus, future studies should analyze the pressure response during cluster designs with an intensity of effort around 30–40% (i.e., 30–40% out of the total feasible repetitions in a set), while allowing long rests between sets [13]. In our study, the increase in SBP during the first set was estimated at an increase of ~7 mmHg per repetition after the forth one, with every new repetition. Additionally, the intersubject variability predicted individual increasing profiles from null to 15 mmHg. In this regard, the development of regression studies will help to anticipate the increase in the pressure response. It will also help stop the set when necessary, in order to reduce the risk of aneurysm rupture. This is when training with continuous designs is needed, such as for eliciting a hypotensive effect [22]. On the other hand, while it seems that IRD might be the best option, when the aim of the resistance training session is to guarantee the electrical integrity of the heart [22], it may not be the best option when trying to maintain the lowest pressure response possible during the exercise. In this particular case, a set that ends around the nadir of the v-shape response would be the predilected option.

This study is not without limitations. Even if efforts were made to obtain appropriate hemodynamic values, data were lost in some participants during the IRD. When leg press was the preferred exercise with intraarterial catheterization [6], it may not be the best exercise with photoplethysmography, since loss of data has been already reported [8]. A better option may be the leg extension exercise since loss of data was not previously informed [16,17]. Besides, while metabolism involvement and intrathoracic pressure were not tested, an inference based on previous studies, describing the response during a set, allowed us a rational explanation of the differences. Lastly, the implementation of our results on the weight room should still be carried out with caution, since, in our analysis, we chose the blood pressure peaks of every repetition. This differs from previous literature, in which analyses were performed considering all beats of every repetition [7,16,17].

## 5. Conclusions

An IRD produced an increase of SBP in the mean of the SBP peaks during the 40 repetitions in comparison with a CD, due to the lack of the v-shape advantage during the former. The increase in SBP was predictable with a mixed linear model during the first set of the CD, with a progressive increase once the nadir was reached during the third repetition. While none of the designs may help to have the actual lowest pressure response possible, these study findings will help to better control the SBP response during a set in resistance exercise.

## Figures and Tables

**Table 1:** Systolic blood pressure (mmHg) responses during baseline and the sessions (*n* = 9).

	Baseline	During Exercise	
		Mean of Peaks ^1^	Peak of the Session ^2^
Interrepetition rest design	112 ± 12	162 ± 21 ^†,‡^	231 ± 24
Continuous design	113 ± 5	148 ± 19 ^†^	228 ± 30

^1^ Mean of the systolic blood pressure peaks of every repetition for the 40 repetitions; ^2^ Peak of systolic blood pressure during the session; ^†^ Interaction reporting a significant difference versus baseline values (*p* < 0.001); ^‡^ Interaction reporting a significant difference versus continuous design (*p* = 0.013). Data are mean ± standard deviation.

**Table 2:** Hemodynamic responses for heart rate, diastolic blood pressure, pulse pressure, and double product during baseline and the sessions (*n* = 9).

	Baseline	During Exercise ^1^
	Heart rate (bpm)	
Interrepetition rest design	54 ± 10	102 ± 20 ^†,‡^
Continuous design	54 ± 10	121 ± 18 ^†^
	Diastolic blood pressure (mmHg)	
Interrepetition rest design	67 ± 7	106 ± 13
Continuous design	67 ± 7	104 ± 14
	Pulse pressure (mmHg)	
Interrepetition rest design	46 ± 7	57 ± 11 ^†,‡^
Continuous design	46 ± 6	43 ± 8
	Double product (bpm × mmHg × 10^−2^)	
Interrepetition rest design	61.8 ± 15	167.3 ± 44.5
Continuous design	60.7 ± 13.1	180.6 ± 41.5

^1^ Mean for the 40 repetitions concurrent with the same cardiac beat as the systolic blood pressure peak of every repetition; ^†^ Significantly different versus baseline values for heart rate (*p* < 0.001) and pulse pressure (*p* = 0.017); ^‡^ Significantly different versus continuous design (*p* < 0.001). Data are mean ± standard deviation.
